# Prognostic value of PD-L1 in esophageal squamous cell carcinoma: a meta-analysis

**DOI:** 10.18632/oncotarget.23810

**Published:** 2017-12-27

**Authors:** Wei Guo, Pan Wang, Ning Li, Fei Shao, Hao Zhang, Zhenlin Yang, Renda Li, Yibo Gao, Jie He

**Affiliations:** ^1^ Department of Thoracic Surgery, National Cancer Center/Cancer Hospital, Chinese Academy of Medical Sciences and Peking Union Medical College, Beijing 10021, The People’s Republic of China

**Keywords:** esophageal squamous cell carcinoma, prognostic value, programmed death receptor 1, programmed death receptor 1 ligand 1, meta-analysis

## Abstract

Accumulated evidence has shown that the programmed cell death receptor 1/programmed cell death receptor 1 ligand 1 (PD-1/PD-L1) pathway is a promising therapeutic target for cancer immunotherapy. However, the association between PD-L1 and esophageal squamous cell carcinoma (ESCC) patient survival remains unclear. We performed a meta-analysis to investigate the prognostic value of PD-L1 in ESCC. We searched PubMed, Embase, Web of Knowledge, and Cochrane Central Register of Controlled Trials databases for relevant studies that evaluated PD-L1 expression and ESCC patient survival. Fixed- and random-effects meta-analyses were conducted according to the heterogeneity of the included studies. Sensitivity analysis was performed according to Metan-based influence analysis. Publication bias was evaluated using Egger’s and Begg’s tests. Overall, 13 studies with 2,877 patients were included. Twelve studies demonstrated the association between overall survival (OS), and 6 studies described the relation between disease-free survival (DFS). PD-L1 overexpression was found in 43.7% (1,258 of 2,877) of the patients with ESCC. High PD-L1 expression was associated with distant metastasis in patients with ESCC (*P =* 0.04). Moreover, high PD-L1 expression was significantly associated with poor OS (hazard ratio [HR] 1.38, 95% confidence interval [CI] = 1.02–1.86, *P =* 0.04) and especially in Asian populations (HR 1.49, 95% CI = 1.11–1.99, *P =* 0.008). But it did not have an impact on disease-free survival (HR 1.15, 95% CI = 0.76–1.74, *P =* 0.52). Further well-designed clinical studies with uniform assessment approaches for PD-L1 expression are warranted to verify its prognostic value.

## INTRODUCTION

Esophageal cancer is the sixth leading cause of cancer-related mortality and the eighth most common cancer worldwide [1]. Esophageal cancer has the following two main subtypes: esophageal squamous cell carcinoma (ESCC) and esophageal adenocarcinoma [2]. In China, esophageal cancer is the fourth leading cause of cancer-related mortality, with ESCC accounting for more than 90% of esophageal cancer cases [2, 3]. Despite clinical advances in radio-chemotherapy and targeted therapy, the 5-year survival rate has been reported to be < 20% [2]. Therefore, it is imperative for researchers to identify precise biomarkers of ESCC and potential therapeutic targets for the disease.

Programmed cell death receptor 1 (PD-1, CD279), which belongs to the B7-CD28 co-stimulatory factor superfamily, is a receptor expressed on the surface of T, B, and Natural killer (NK) cells that regulate their activation and apoptosis [4]. Its ligand, programmed death receptor 1 ligand 1 (PD-L1, CD274, B7-H1), is expressed on cancer cells and immune cells and plays a crucial role in blocking the “cancer immunity cycle” [5]. Binding of PD-L1 to PD-1 suppresses T-cell migration, proliferation, and secretion of cytotoxic mediators, and restricts cancer cell death [6–8]. Moreover, blockade of the PD-1/PD-L1 pathway with monoclonal antibodies (against PD-1 or PD-L1) has shown promising results for several types of human cancers [9–12].

PD-L1 overexpression has been observed in various types of solid tumors, including melanoma, lung cancer, breast cancer, colorectal cancer, bladder cancer, gastric cancer, hepatocellular carcinoma, renal cell carcinoma, papillary thyroid cancer, and head and neck cancer [13–23]. A meta-analysis of 28 studies with 3,107 patients having various solid malignancies demonstrated that PD-L1 overexpression was associated with poor overall survival (OS) [24]. Recently, several meta-analyses demonstrated that PD-L1 overexpression was associated with poor prognosis in many cancer types [25–28]. On the contrary, PD-L1 overexpression was found to be associated with better prognosis in non-small cell lung cancer (NSCLC), colorectal cancer, pancreatic cancer, breast cancer, and Merkel cell carcinoma [29–34].

However, in ESCC, the number of studies are limited and the prognostic value of PD-L1 expression still remains controversial, as some studies associate PD-L1 expression with a rather favorable prognosis, while others postulate a less favorable disease course for cancers with high PD-L1 expression [35–37].

To address this issue, we conducted a meta-analysis to investigate the correlation between PD-L1 overexpression and ESCC prognosis.

## RESULTS

### Search results and study characteristics

**Figure 1:**
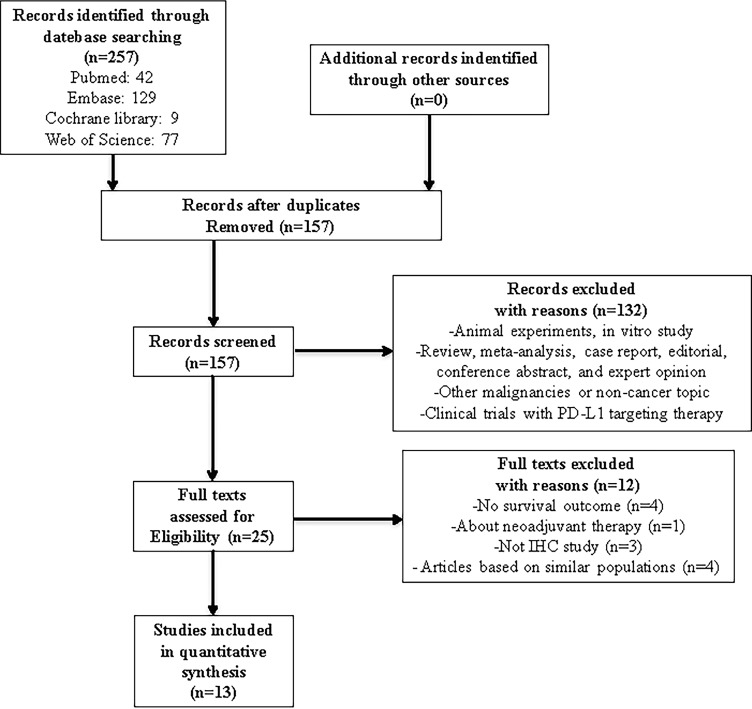
Flowchart of articles reviewed and included in our meta-analysis

In this study, we identified a total of 257 potentially relevant articles with our search strategy. After screening the titles and abstracts of these articles, we excluded 232 studies because they were duplicate studies or were irrelevant. After reading 25 potentially eligible articles in detail, we finally included 13 studies in this meta-analysis [36–48]. A detailed flowchart of the above screening process is presented in Figure 1.

The characteristics of the included studies are presented in Table 1. The sample sizes of these studies ranged from 90 to 536 patients, and a total of 2,877 patients were enrolled in these studies. All 13 included studies were retrospective. Of the 13 studies, eight originated from China and the remaining five originated from Japan, Germany, and South Korea.

**Table 1:** Characteristics of studies included in the meta-analysis

First Author	Year	Country	No. of patients	Age Median (range) (years)	IHC evaluation method	Antibody				Cutoff	PD-L1 positive (%)	Follow-up Median (range) (months)	Quality Assessment (score)
						Company	Source	Clone	Dilution				
Chen, K	2016	China	536	63 (46–73)	Percentage	Sigma-Aldrich	Rabbit	SAB2900365	1:400	≥5%	41.4% (222/536)	32.7 (1.0–88.7)	7
Chen, L	2014	China	99	59	H-score	Novus Biologicals	Rabbit	NBP1-03220	1:200	>0	82.8% (82/99)	NA	7
Chen, MF	2016	China (Taiwan)	162	NA	IRS score	R&D Systems or Biolegend	NA	NA	NA	≥ 2	45% (74/162)	NA	6
Hatogai	2016	Japan	196	65 (42–87)	Percentage	Cell Signaling Technology	Rabbit	E1L3N	1:400	≥ 1%	18.4% (36/196)	66 (1.2–127.2)	8
Jesingha-us	2017	Germany	125	60 (39–83)	Percentage	NA	NA	SP263	1:400	≥ 10%	30.4% (38/125)	65.09	8
Jiang, D	2017	China	278	62 (37–82)	Percentage	OriGene Technologies	Rabbit	SP142	1:300	≥ 5%	45% (125/278)	33 (2–102)	8
Jiang, Y Cohort A	2016	China	250	NA	Percentage	Merck KGaA	Rabbit	73-10	1:1000	≥ 1%	78.4% (196/250)	34.4 (0.3–147.1)	7
Jiang, Y Cohort B	2016	China	78	NA	Percentage	Merck KGaA	Rabbit	73-10	1:1000	≥ 1%	80.8% (63/78)	34.4 (0.3–147.1)	7
Kim	2016	South Korea	200	65 (41–83)	H-score	Cell Signaling Technology	Rabbit	E6H4	NA	≥ 1	33.5 (67/200)	33.2 (0.6–178.7)	8
Leng	2016	China	106	59 (38–80)	H-score	Abcam	Rabbit	ab58810	1:40	≥ 3	46.2% (49/106)	55	6
Tanaka	2016	Japan	180	64 (29–84)	IRS score	Woburn	mouse	27A2	NA	≥ 4	29.4% (53/180)	24 (1–196)	7
Tsutsumi	2017	Japan	90	62.7	Percentage	Lifespan Bioscience	Rabbit	NA	1:200	> 5%	63.3% (57/90)	NA	6
Zhang	2017	China	344	NA	Percentage	Spring Bioscience	Rabbit	SP142	NA	> 5%	14.5% (50/344)	NA	6
Zhu	2017	China	133	NA	Percentage	Beijing Zhongshan Golden Bridge Biotechnology	Rabbit	SP142	NA	> 5%	51.1% (68/133)	42.6	6

All the studies performed immunohistochemical (IHC) analysis to evaluate PD-L1 expression in ESCC tissues, with PD-L1 positivity rates ranged from 18.4% to 82.8%. The hazard ratios (HRs) and 95% confidence intervals (CIs) were obtained from the original text or from the original authors. Among the 13 studies, 12 studies demonstrated the association between overall survival (OS) and PD-L1 expression, and 6 studies described the relationship between disease-free survival (DFS) and PD-L1 expression. The Newcastle-Ottawa quality assessment scale (NOS) score for study quality ranged from 6 to 8 [49].

### Association between PD-L1 expression and OS

**Figure 2:**
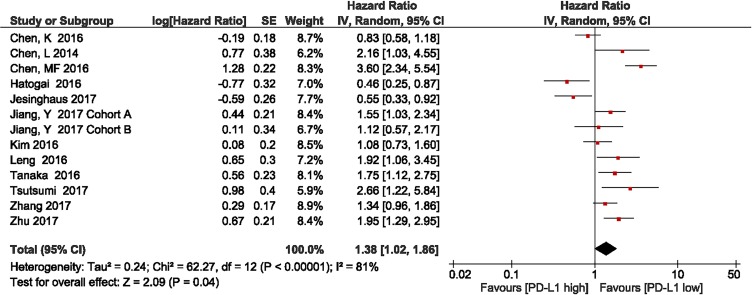
Forest plot describing the association between PD-L1 expression and overall survival in patients with esophageal squamous cell carcinoma

We investigated the association between PD-L1 expression and OS in patients with ESCC. Twelve studies with a total of 2,499 patients were included. The meta-analysis showed that among patients with ESCC, PD-L1 overexpression was associated with shorter OS compared with the finding in patients with low PD-L1 expression (HR = 1.38, 95% CI 1.02–1.86; *P* = 0.04). Significant heterogeneity was observed (*I*^2^ = 80%, *P* < 0.00001); therefore, a random effects model was used for the analysis (Figure 2).

### Association between PD-L1 expression and disease-free survival

**Figure 3:**
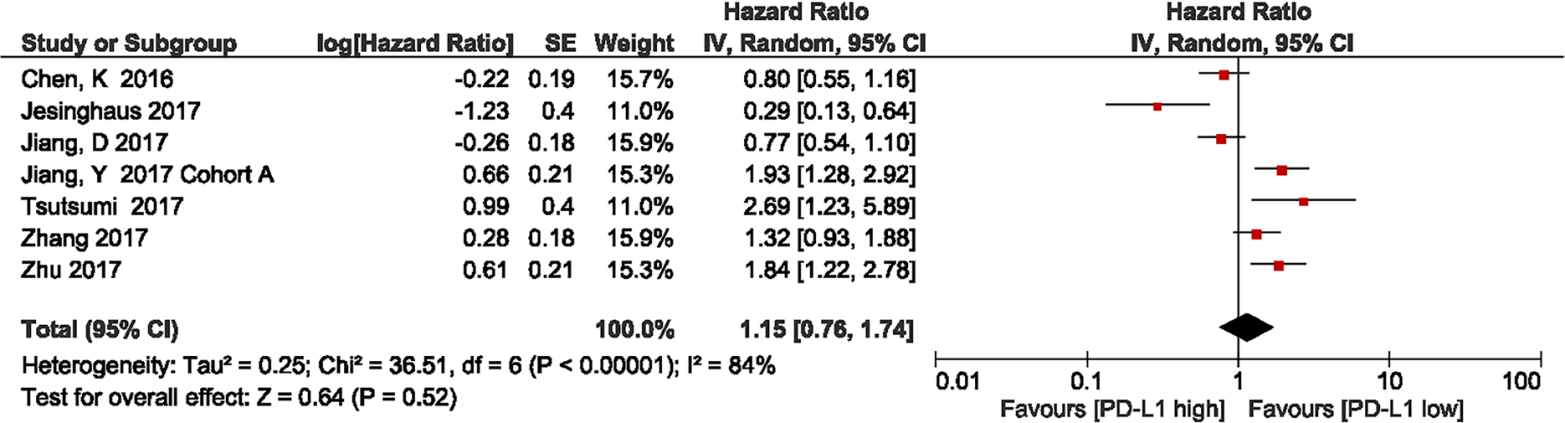
Forest plot describing the association between PD-L1 expression and disease-free survival in patients with esophageal squamous cell carcinoma

We investigated the association between PD-L1 expression and DFS in patients with ESCC. Six studies with a total of 1,756 patients were included. The meta-analysis showed that among patients with ESCC, PD-L1 overexpression was associated with shorter DFS compared with the finding in patients with low PD-L1 expression, but there was no statistical significance (HR = 1.15, 95% CI 0.76–1.74; *P* = 0.52). Significant heterogeneity was observed (*I*^2^ = 84%, *P* < 0.00001); therefore, a random effects model was used for the analysis (Figure 3).

### Association between PD-L1 expression and clinicopathological characteristics

We investigated the association between PD-L1 expression and the clinicopathological characteristics of patients with ESCC.

High PD-L1 expression was found in 43.7% (1,258 of 2,877) of patients with ESCC. Pooled results showed that PD-L1 expression was high in patients with distant metastasis (odds ratio [OR] = 1.58, 95% CI 1.03–2.42; *P* = 0.04). However, we detected no significant associations between PD-L1 overexpression and sex (OR = 0.92, 95% CI 0.73–1.16; *P* = 0.48), T stage (OR = 0.96, 95% CI 0.60–1.53; *P* = 0.86), N stage (OR = 1.26, 95% CI 0.80–2.00; *P* = 0.31), TNM stage (OR = 0.99, 95% CI 0.72–1.38; *P* = 0.97), tumor grade (OR = 1.01, 95% CI 0.66–1.54; *P* = 0.95), lymphatic invasion (OR = 1.15, 95% CI 0.81–1.65; *P* = 0.44), venous invasion (OR = 1.06, 95% CI 0.57–2.92; *P* = 0.80), and neoadjuvant treatment (OR = 1.28, 95% CI 0.57–2.92; *P* = 0.55). Moreover, there were no significant associations between PD-L1 expression and drinking (OR = 0.89, 95% CI 0.63–1.27; *P* = 0.53) or smoking history (OR = 0.86, 95% CI 0.64–1.14; *P* = 0.30; Table 2 and supplementary materials).

**Table 2:** Association between PD-L1 expression and clinicopathological characteristics

Clinicopathological feature	Studies	Heterogeneity		OR (95% CI)	*P*-value
		*P*-value	*I*^2^ (%)		
Sex	11	0.60	0	0.92 (0.73–1.16)	0.48
Differentiation	9	0.002	68	1.01 (0.66–1.54)	0.95
T stage	6	0.02	64	0.96 (0.60–1.53)	0.86
N stage	9	< 0.00001	79	1.26 (0.80–2.00)	0.31
Metastasis	4	0.97	0	1.58 (1.03–2.42)	0.04
TNM stage	8	0.02	57	0.99 (0.72–1.38)	0.97
Lymphatic invasion	3	0.20	37	1.15 (0.81–1.65)	0.44
Venous invasion	3	0.22	34	1.06 (0.67–1.67)	0.80
Neoadjuvant treatment	3	0.04	70	1.28 (0.57–2.92)	0.55
Drinking	2	0.30	5	0.89 (0.63–1.27)	0.53
Smoking	3	0.65	0	0.86 (0.64–1.14)	0.30

Heterogeneity was not observed in the analysis of the relationships between PD-L1 expression and sex (*P* = 0.60, *I*^2^ = 0), lymphatic invasion (*P* = 0.20, *I*^2^ = 37%), venous invasion (*P* = 0.22, *I*^2^ = 34%), metastasis (*P* = 0.97, *I*^2^ = 0), and smoking history (*P* = 0.65, *I*^2^ = 0); therefore, a fixed effects model was used. The other assessments were performed using a random effects model (Table 2).

### Subgroup and sensitivity analysis

**Figure 4:**
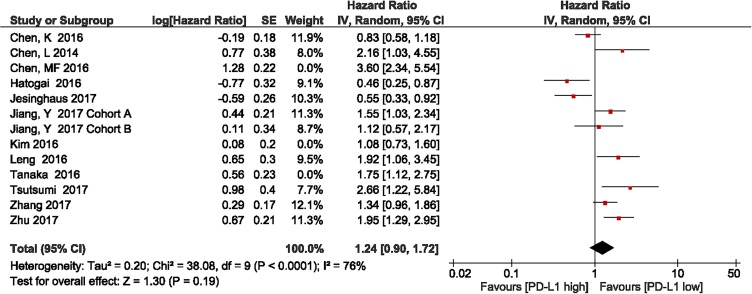
Forest plot describing subgroup analysis of the association between PD-L1 expression and overall survival after removal of the studies by Chen et al., Kim et al., and Tanaka et al

The studies by Chen et al., Kim et al., and Tanaka et al. included patients who had received neoadjuvant treatment [42, 45, 47]. The subgroup meta-analysis of the 9 studies without neoadjuvant treatment showed that patients with high PD-L1 expression had shorter OS, compared with those with low PD-L1 expression (HR = 1.24, 95% CI 0.90–1.72; *P* = 0.19, Figure 4). Heterogeneity was observed in this subgroup analysis (*I*^2^ = 76%, *P* < 0.0001); therefore, a random effects model was used.

**Figure 5:**
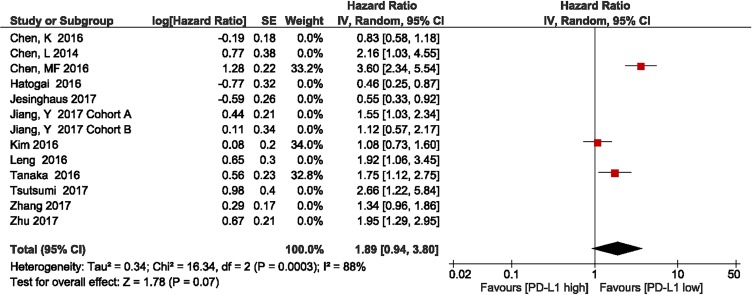
Forest plot describing subgroup analysis of the association between PD-L1 expression and overall survival in the studies by Chen et al., Kim et al., and Tanaka et al

The subgroup analysis of the three studies showed that patients with high PD-L1 expression had shorter OS, compared with those with low PD-L1 expression (HR = 1.89, 95% CI 0.94–3.80; *P* = 0.07; Figure 5). Heterogeneity was observed in this subgroup analysis (*I*^2^ = 88%, *P* = 0.0003); therefore, a random effects model was used.

**Figure 6:**
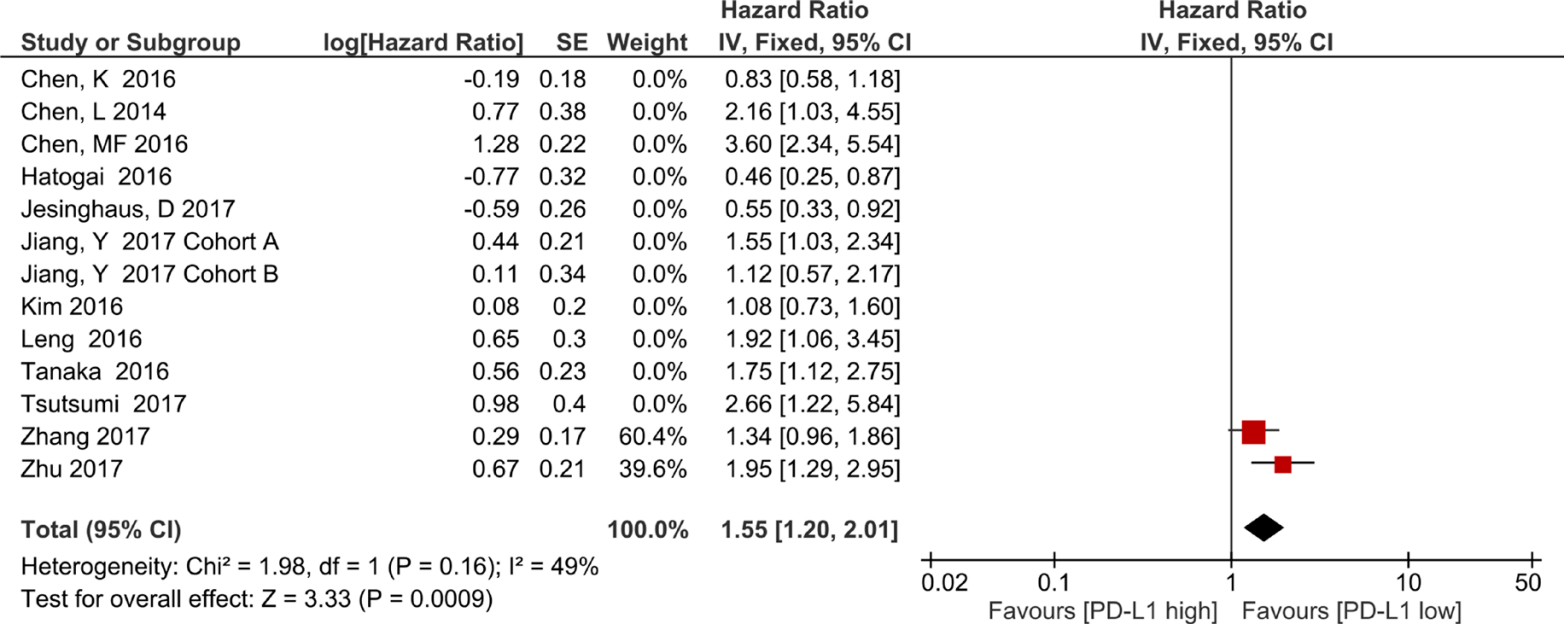
Forest plot describing subgroup analysis of the association between PD-L1 expression and overall survival in studies with the same anti-PD-L1 antibody and cutoff value

The subgroup meta-analysis of two studies with the same PD-L1 antibody and cutoff value, the result showed OS was significantly associated with PD-L1 overexpression (HR = 1.55, 95% CI 1.20–2.01; *P* = 0.0009, Figure 6). Heterogeneity was not observed in this subgroup analysis (*I*^2^ = 49%, *P* < 0.16); therefore, a fixed effects model was used.

**Figure 7:**
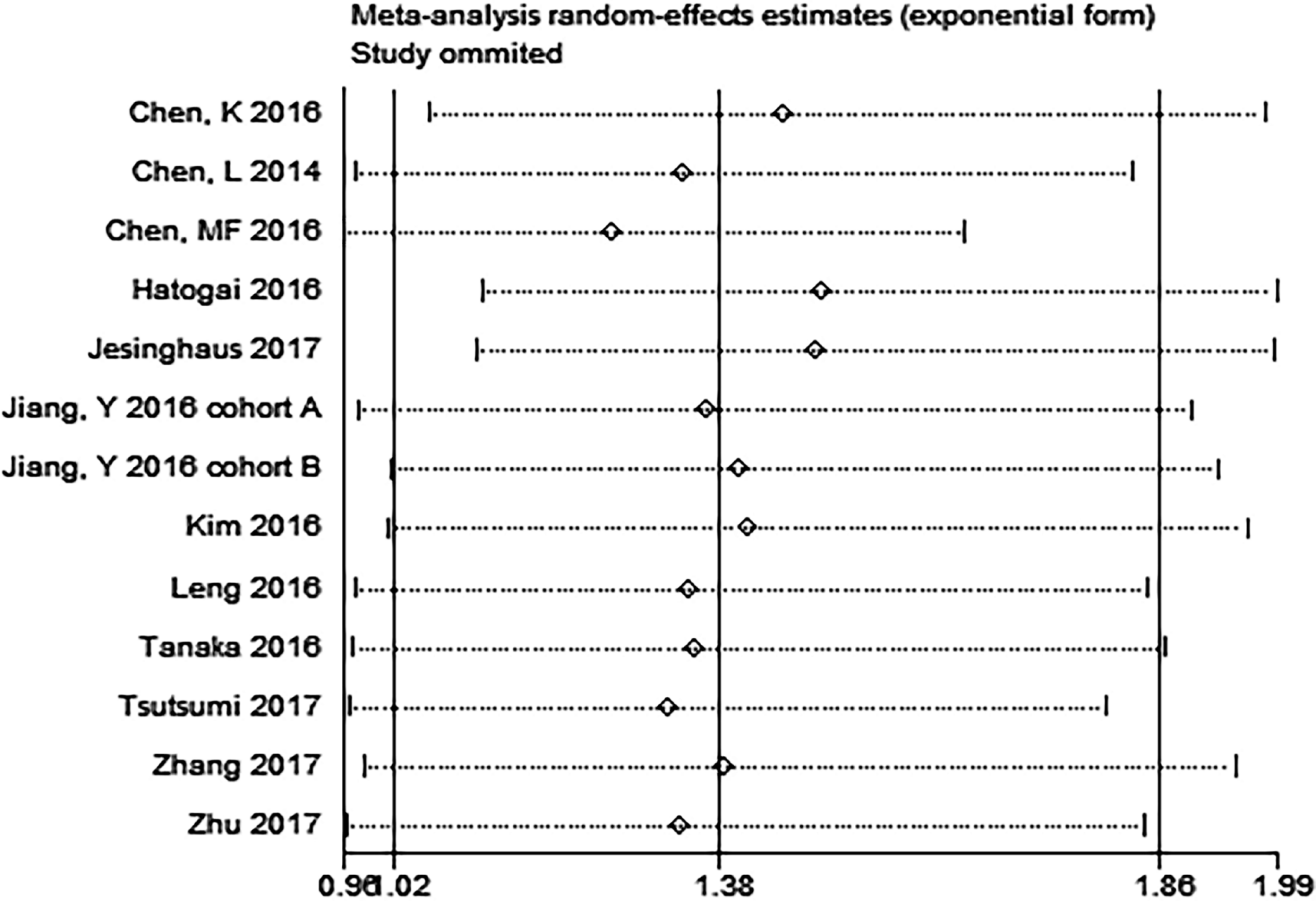
Metan-based influence analysis of the hazard ratios of overall survival

**Figure 8:**
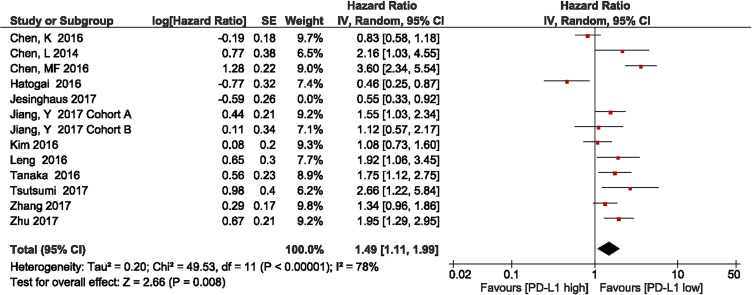
Forest plot describing subgroup analysis of the association between PD-L1 expression and overall survival in Asian population studies after removal of the study by Jesinghaus et al

Metan-based influence analysis (Stata; Stata Corporation, Texas, USA) was performed to evaluate the stability of the results. The results of the analysis demonstrated that no individual study significantly influenced the HRs of OS, suggesting that the results of the present meta-analysis are credible (Figure 7). Additionally, the study by Jesinghaus et al. [46] included Non-Asian patients. Removal of this study enhanced the association between PD-L1 expression and OS (HR = 1.49, 95% CI 1.11–1.99; *P* = 0.008; Figure 8).

### Publication bias

**Figure 9:**
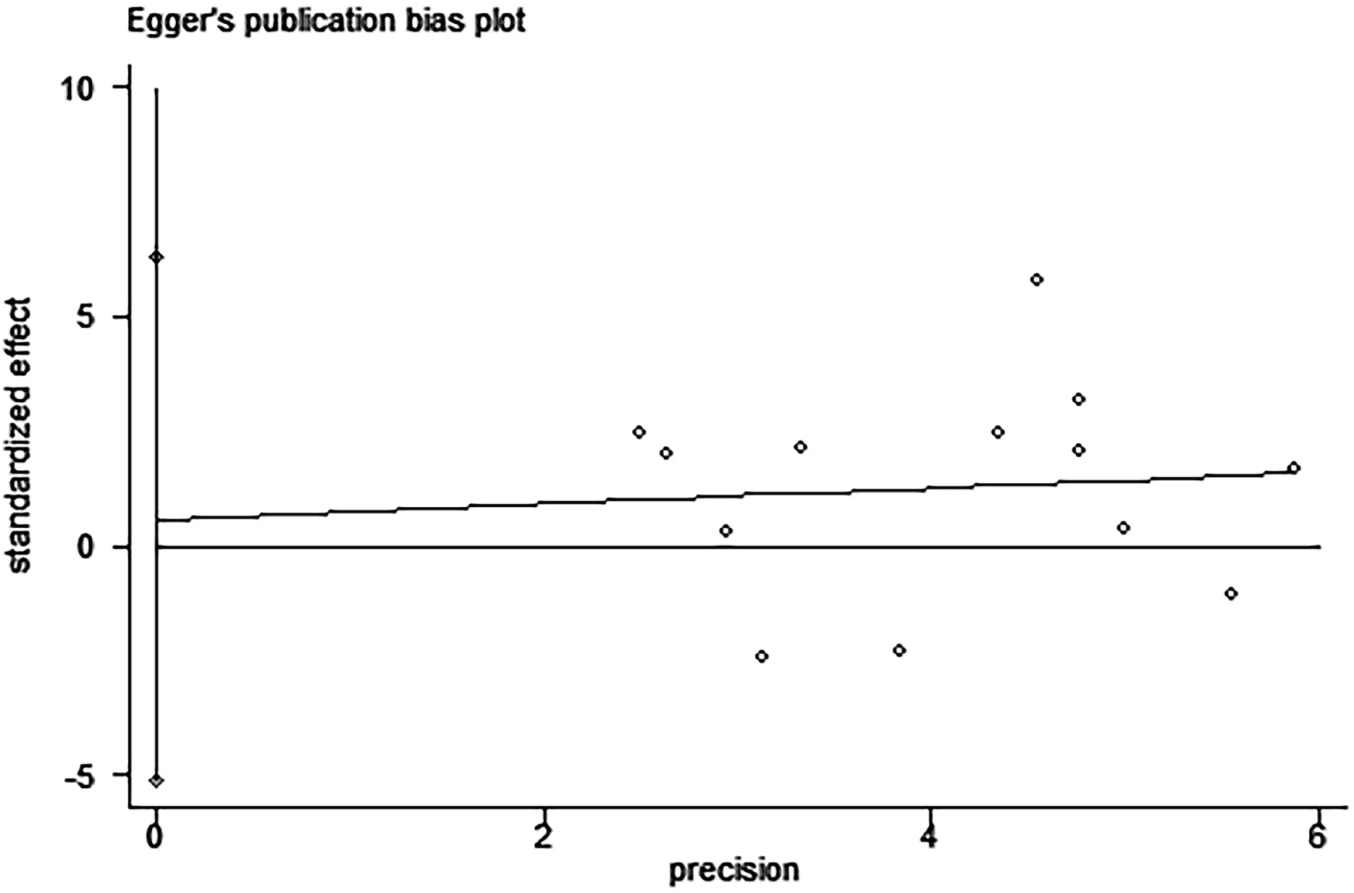
Egger’s test for the assessment of potential publication bias in studies investigating the association between PD-L1 expression and overall survival of patients with esophageal squamous cell carcinoma Egger’s test shows no evidence of publication bias (Egger’s *P* = 0.822) among the studies reporting the outcome of overall survival.

**Figure 10:**
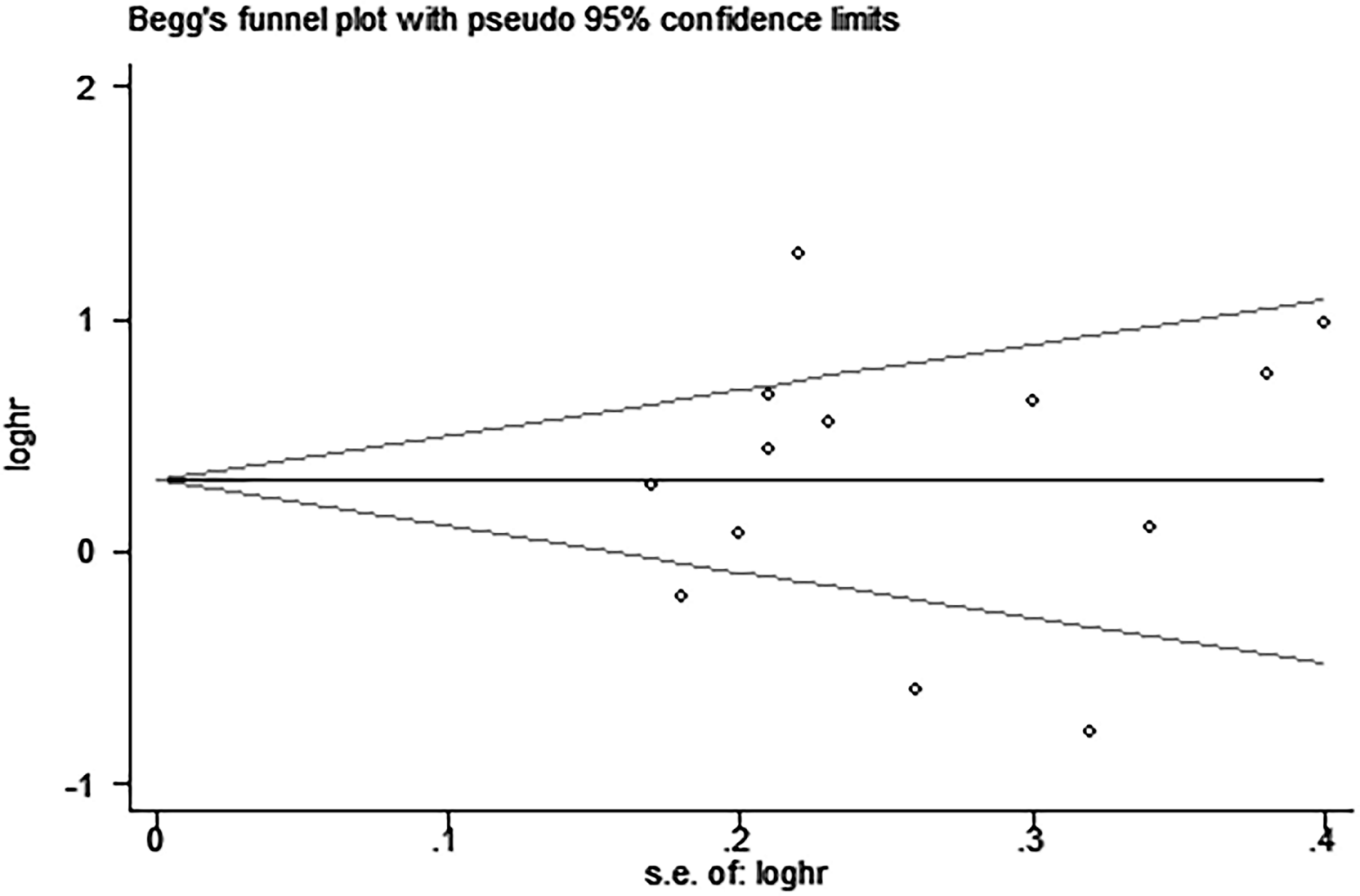
Begg’s funnel plot for the assessment of potential publication bias in studies investigating the association between PD-L1 expression and overall survival of patients with esophageal squamous cell carcinoma No evidence of publication bias is observed, as indicated by a symmetric funnel plot (Begg’s *P* = 0.392).

**Figure 11:**
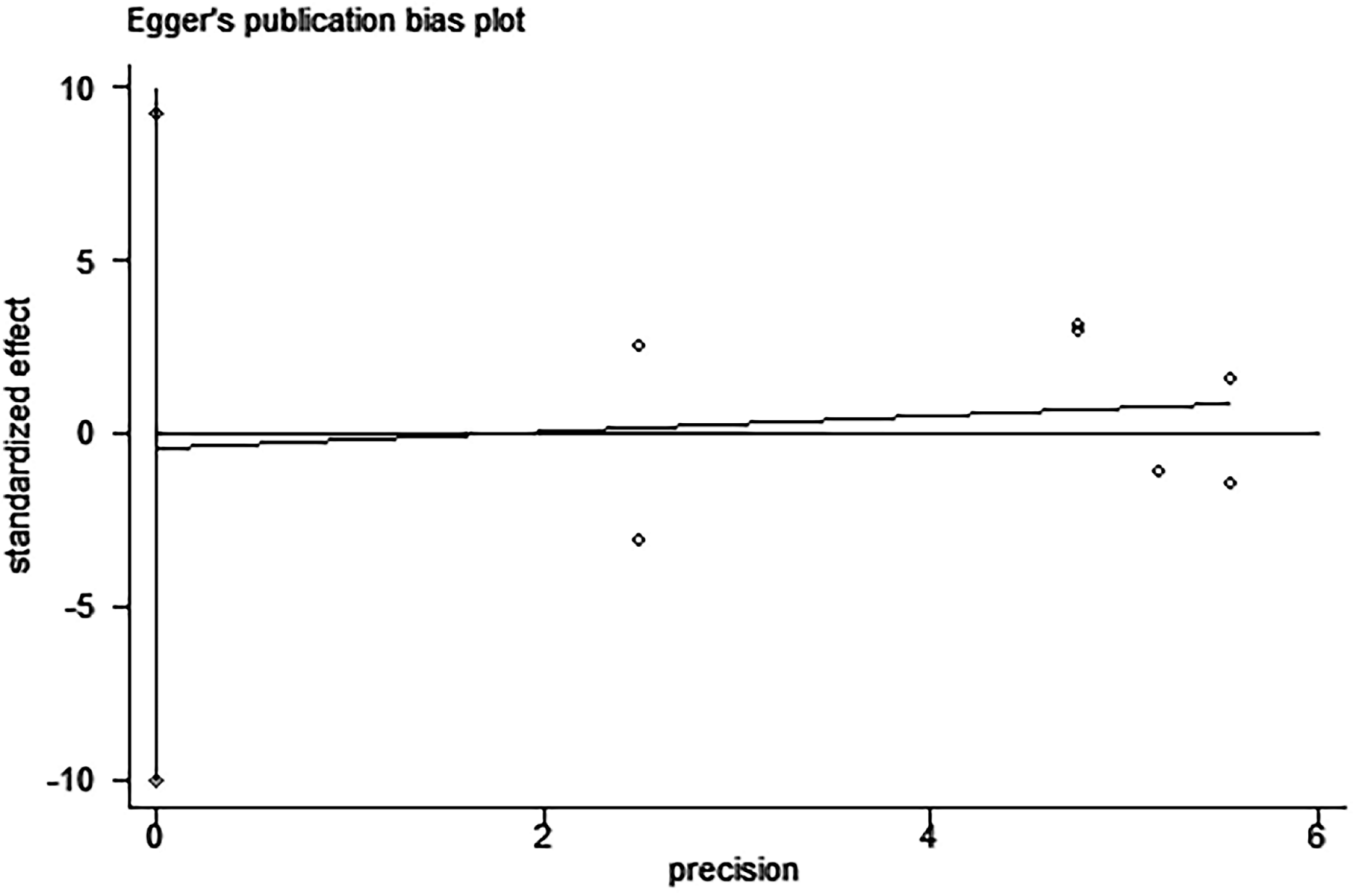
Egger’s test for the assessment of potential publication bias in studies investigating the association between PD-L1 expression and disease-free survival of patients with esophageal squamous cell carcinoma Egger’s test shows no evidence of publication bias (Egger’s *P* = 0.917) among the studies reporting the outcome of disease-free survival.

**Figure 12:**
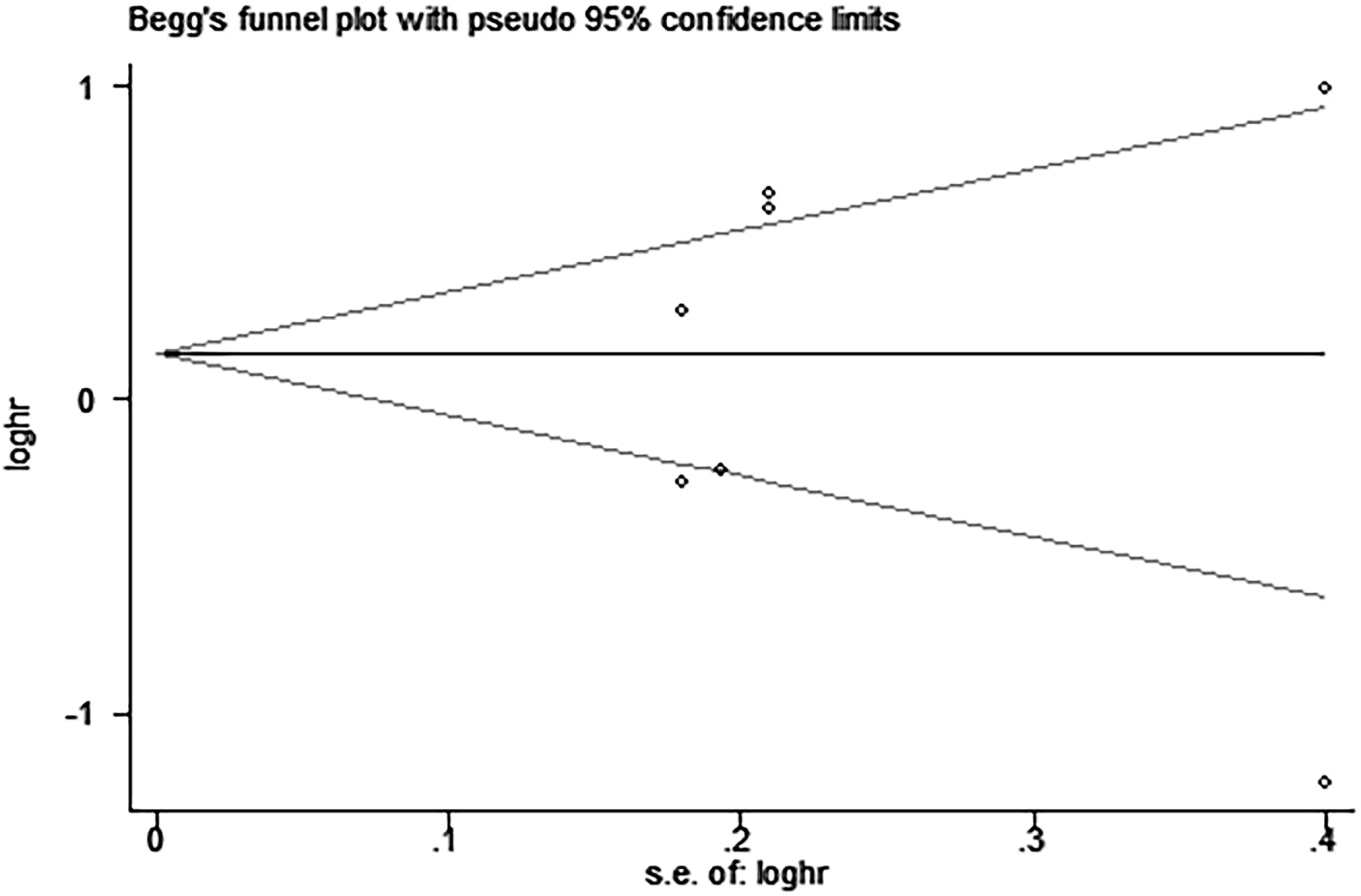
Begg’s funnel plot for the assessment of potential publication bias in studies investigating the association between PD-L1 expression and disease-free survival of patients with esophageal squamous cell carcinoma No evidence of publication bias is observed, as indicated by a symmetric funnel plot (Begg’s *P* = 0.876).

Egger’s and Begg’s tests indicated that no publication bias affected the HRs for OS and DFS. The *P*-values for these tests were 0.822 and 0.392 (OS) and 0.917 and 0.876 (DFS), respectively (Figures 9–12).

## DISCUSSION

Recently, many researchers have focused their attention on PD-L1 expression in various solid tumor, due to the FDA’s approval for anti-PD-L1 therapy in several kinds of cancer with good efficacy and safety [50]. Several clinical trials have reported that these immune checkpoint therapies improved patients’ outcomes, while tumor response has been related to PD-L1 expression [51–53]. PD-L1 overexpression has been reported in various cancer, and a previous meta-analysis demonstrated that high PD-L1 expression was associated with poor OS in human solid tumors [24]. However, the relationship between PD-L1 expression and the prognosis of patients with ESCC remains unclear. Multiple studies have indicated that PD-L1 expression is associated with a significant poor survival outcome [38, 39, 42, 44, 45, 48], while two studies reported the opposite effect [40, 46], and the other studies have shown no association [36, 37, 43, 47].

A meta-analysis by Qu et al. published in 2016 demonstrated that high PD-L1 expression might impair the prognosis of ESCC, but the finding was not statistically significant [28]. Our meta-analysis included 13 studies with 2,777 patients and illustrated that high PD-L1 expression was associated with distant metastasis and poor OS, but not with tumor grade, TNM stage, lymph node metastasis, neoadjuvant treatment and DFS. The two differences between our meta-analysis and the previous one were: 1) In our meta-analysis, we added 6 studies by Jesinghaus et al, Jiang et al, Kim et al, Tsutusmi et al, Zhang et al and Zhu et al [36, 39, 43, 44, 46, 47], which were published durig 2016–2017; 2) All survival data in our meta-analysis were directly extracted from tables or text of the included studies or were obtained by contacting the original authors. However, in Qu’s analysis, some survival data were calculated or estimated from Kaplan-Meier curves, which may have compromised the precision of the data. These two differences, to some extent, cause our different results.

In our meta-analysis, PD-L1 overexpression was associated with distant metastases and OS, while it had no significant impact on DFS. The possible reason was the limited number of studies included when performing the analysis between PD-L1 overexpression and DFS.

In our subgroup meta-analysis of the 9 studies without neoadjuvant chemotherapy showed that patients with high PD-L1 expression had shorter OS, compared with those with low PD-L1 expression. And for the remaining three studies with neoadjuvant chemotherapy showed that PD-L1 overexpression is associated with shorter OS in ESCC. However, both of them had no statistical significance. The possible reason is the limited number of studies included and the lack of uniform standardization for PD-L1 assessment. And another subgroup analysis followed was to evaluate the impact of different PD-L1 assessment on survival results. In the two studies with the same antibody and cutoff value, the result showed OS was significantly associated with PD-L1 overexpression. Moreover, heterogeneity was not observed in this subgroup analysis.

Moreover, in metan-based influence analysis demonstrated that no individual study significantly influenced the HRs of OS, suggesting that the results of the present meta-analysis are credible. Moreover, removal of the only one non-Asian study by Jesinghaus et al. enhanced the association between PD-L1 expression and OS (HR = 1.49, 95% CI 1.11–1.99; *P* = 0.008). The possible reason for this finding is racial difference.

Theoretically, the interaction between PD-L1 in tumor cells and PD-1 in T cells negatively regulates the tumor-killing function of T-cells and protects tumor cells from the host immune system. As for ESCC, *in vitro* studies demonstrated that the count of PD-1 positive TILs (tumor-infiltrating lymphocytes) was negatively correlated with PD-L1 expression. High PD-L1 expression in cancer cells might prevent effective antitumor immunity [48]. Tsutsumi et al. also reported that PD-L1 expression at the invasive front of ESCC was related to epithelial-mesenchymal transition (EMT). And there might be a cooperative mechanism between tumor immune avoidance and EMT contributes to tumor malignancy [44]. Taken together, ESCC cancer cells with high PD-L1 expression should be more invasive.

However, comparisons of different studies reporting PD-L1 expression in ESCC are possibly hindered by the use of different tricks of immunohistochemical technology. Furthermore, cutoff value used for assessing PD-L1 expression may lack sensitivity and yield false-negative results, and there is no uniform standard at present. Different anti-PD-L1 antibodies and specimens from different areas may also lead to different results. In additional, the expression of PD-L1 is dynamic, and it might also lead to false negative results [54].

We made an effort to conduct a comprehensive analysis, but some limitations should be acknowledged. First, our meta-analysis was limited to articles published in English. Second, most included studies were performed in East Asia. Our results should be confirmed in a wider range of populations, especially in Western countries. Third, the sample sizes of some included studies were relatively small, although the results of the sensitivity analysis remained stable after the sequential exclusion of each individual study. Finally, no standardization was present with regard to the methodology of PD-L1 assessment among the studies included in our analysis, which may have caused great heterogeneity among the studies. Despite these limitations, this meta-analysis demonstrated associations between PD-L1 expression and clinicopathological factors of ESCC.

In summary, our meta-analysis indicated that high PD-L1 expression in ESCC was associated with distant metastasis and reduced OS. However, the findings need to be confirmed in future adequately designed clinical studies with uniform assessment approaches.

## MATERIALS AND METHODS

### Search strategy

We performed a comprehensive literature search for published articles using the PubMed, Embase, Web of Knowledge, and Cochrane Central Register of Controlled Trials databases. Articles published before July 2017 were included in this analysis.

The following medical subject headings and keywords were used for the search: “Esophageal Neoplasms” [Mesh], “Antigens, CD274” [Mesh], “Esophageal Neoplasm,” “Neoplasm, Esophageal,” “Esophagus Neoplasm,” “Esophagus Neoplasms,” “Neoplasm, Esophagus,” “Neoplasms, Esophagus,” “Neoplasms, Esophageal,” “Cancer of Esophagus,” “Cancer of the Esophagus,” “Esophagus Cancer,” “Cancer, Esophagus,” “Cancers, Esophagus,” “Esophagus Cancers,” “Esophageal Cancer,” “Cancer, Esophageal,” “Cancers, Esophageal,” “Esophageal Cancers,” “Esophageal Squamous Cell Carcinoma,” “CD274 Antigens,” “B7-H1 Immune Costimulatory Protein,” “B7 H1 Immune Costimulatory Protein,” “B7-H1 Antigen,” “Antigen, B7-H1,” “B7 H1 Antigen,” “PD-L1 Costimulatory Protein,” “Costimulatory Protein, PD-L1,” “PD L1 Costimulatory Protein,” “Programmed Cell Death 1 Ligand 1 Protein,” “CD274 Antigen,” “Antigen, CD274,” “Programmed Cell Death 1 Ligand 1,” “B7H1 Immune Costimulatory Protein,” “B7-H1,” “PD-L1,” “B7 H1,” and “CD274.”

The article language was restricted to English. To identify additional studies, we also reviewed the reference lists of relevant articles.

### Selection criteria

The inclusion criteria were as follows: (1) the entire study population comprised patients with histologically confirmed ESCC; (2) PD-L1 protein expression in the primary ESCC tissue was detected by IHC analysis; (3) data regarding the correlation between PD-L1 and clinicopathological parameters were provided; and (4) sufficient survival data were provided to estimate the prognosis. Moreover, if there were multiple articles based on similar populations, only the most recent article was included.

The exclusion criteria were as follows: (1) *in vitro* studies and animal experiments; (2) review, meta-analysis, editorial, case report, conference abstract, and expert opinion; and (3) studies on the PD-L1 level of TIL or circulating tumor cells.

### Data extraction and quality assessment

Eligible reports were identified by two reviewers (Wei Guo and Pan Wang), and disagreements were resolved by a third reviewer (Yibo Gao).

Cohort level characteristics (name of the first author, year of publication, country, number of patients, clinicopathological characteristics of patients, IHC evaluation methods, antibodies, cutoff value to determine PD-L1 positivity, etc.) were extracted for statistical analysis. Survival data (HR, CI, and *P*-value) were directly extracted from tables or text of the included studies or were obtained by contacting the original authors. When both univariate and multivariate analysis for survival outcome were provided, only the multivariate analysis was extracted since it has been more precise.

The NOS was used, and any discrepancies in the score were resolved by discussing the findings and reaching a consensus. The maximum possible score for the NOS is 9 points, and a study that achieved a score of 6 or higher was considered to be of high quality [15].

### Statistical analysis

Pooled ORs and their 95% CIs were analyzed to determine the association between PD-L1 expression and clinicopathological parameters, and HRs and their 95% CIs were used to evaluate the association between PD-L1 expression and survival of patients with ESCC. A *P-*value < 0.05 was considered to indicate statistical significance. Heterogeneity among studies was tested using the chi-square test and I-Square. A *P*-value < 0.1 or *I*^2^ > 50% was considered to indicate significant heterogeneity among studies. If heterogeneity was identified among studies, a random effects model was used to pool the ORs, and if not, a fixed effects model was selected. The potential for publication bias was assessed using both Begg’s rank correlation method and Egger’s weighted regression method [55, 56]. All analyses were performed using Review Manager 5.3 (Cochrane Collaboration, Oxford, UK) and Stata 12.0 (Stata Corporation).

## SUPPLEMENTARY MATERIALS FIGURES



## Abbreviations

PD-1: Programmed cell death receptor 1
PD-L1: Programmed cell death receptor 1 ligand 1
ESCC: Esophageal squamous cell carcinoma
NK cells: Natural killer
NSCLC: Non-small cell lung cancer
DFS: Disease-free survival
OS: Overall survival
HR: Hazard ratio
CI: Confidence interval
IHC: Immunohistochemical
HNSCC: Head and neck squamous cell carcinoma
LSCC: Lung squamous cell carcinoma
LAOCC: Local advanced oral squamous cell carcinoma
NOS: Newcastle-Ottawa Quality Assessment Scale
TILs: Tumor-infiltrating lymphocytes
EMT: Epithelial-mesenchymal transition
