# Supplementary material for: Prognostic value of PD-L1 in esophageal squamous cell carcinoma: a meta-analysis
Source: Oncotarget. 2017 Dec 27;9(17):13920–33. doi: 10.18632/oncotarget.23810 (PMC5862626; doi:10.18632/oncotarget.23810)
Supplement: Supplementary file 1 [file oncotarget-09-13920-s001.pdf]

Prognostic value of PD-L1 in esophageal squamous cell carcinoma: a meta-analysis

SUPPLEMENTARY MATERIALS

Study or Subgroup	Male Events	Male Total	Female Events	Female Total	Weight	Odds Ratio M-H, Fixed, 95% CI
Chen, K 2016	193	464	29	72	19.1%	1.06 [0.64, 1.75]
Chen, L 2014	62	76	20	23	3.7%	0.66 [0.17, 2.55]
Hatogai 2016	26	160	10	36	8.9%	0.50 [0.22, 1.17]
Jiang, D 2017	107	232	18	46	10.5%	1.33 [0.70, 2.54]
Jiang, Y 2017	267	335	74	93	15.3%	1.01 [0.57, 1.78]
Kim 2016	63	188	4	12	3.3%	1.01 [0.29, 3.48]
Leng 2016	42	76	15	30	6.3%	1.24 [0.53, 2.88]
Tanaka 2016	48	157	5	23	3.9%	1.59 [0.56, 4.52]
Tsutsumi 2017	49	80	8	10	3.6%	0.40 [0.08, 1.98]
Zhang 2017	35	268	15	76	13.2%	0.61 [0.31, 1.19]
Zhu 2017	29	75	27	58	12.2%	0.72 [0.36, 1.45]
**Total (95% CI)**		**2111**		**479**	**100.0%**	**0.92 [0.73, 1.16]**
Total events	921		225			

Heterogeneity: Chi² = 8.31, df = 10 (P = 0.60); I² = 0%
Test for overall effect: Z = 0.70 (P = 0.48)

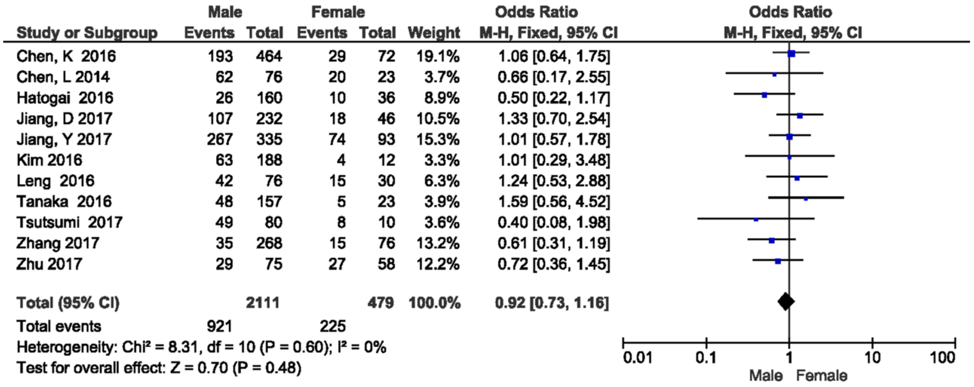

Supplementary Figure 1: Forest plot describing the association between PD-L1 expression and gender.

Study or Subgroup	Tumor grade 3 Events	Tumor grade 3 Total	Tumor grade 1-2 Events	Tumor grade 1-2 Total	Weight	Odds Ratio M-H, Random, 95% CI
Chen, K 2016	38	124	184	412	16.4%	0.55 [0.36, 0.84]
Hatogai 2016	5	21	31	175	8.6%	1.45 [0.49, 4.26]
Jiang, D 2017	49	115	76	163	15.7%	0.85 [0.53, 1.37]
Jiang, Y 2017	128	171	213	257	15.8%	0.61 [0.38, 0.99]
Kim 2016	9	23	58	172	10.5%	1.26 [0.52, 3.09]
Leng 2016	19	37	38	69	11.6%	0.86 [0.39, 1.92]
Tsutsumi 2017	22	17	46	73		Not estimable
Zhang 2017	6	37	44	307	10.1%	1.16 [0.46, 2.93]
Zhu 2017	23	34	33	99	11.2%	4.18 [1.82, 9.60]
**Total (95% CI)**		**579**		**1727**	**100.0%**	**1.01 [0.66, 1.54]**
Total events	299		723			

Heterogeneity: Tau² = 0.23; Chi² = 22.08, df = 7 (P = 0.002); I² = 68%
Test for overall effect: Z = 0.06 (P = 0.95)

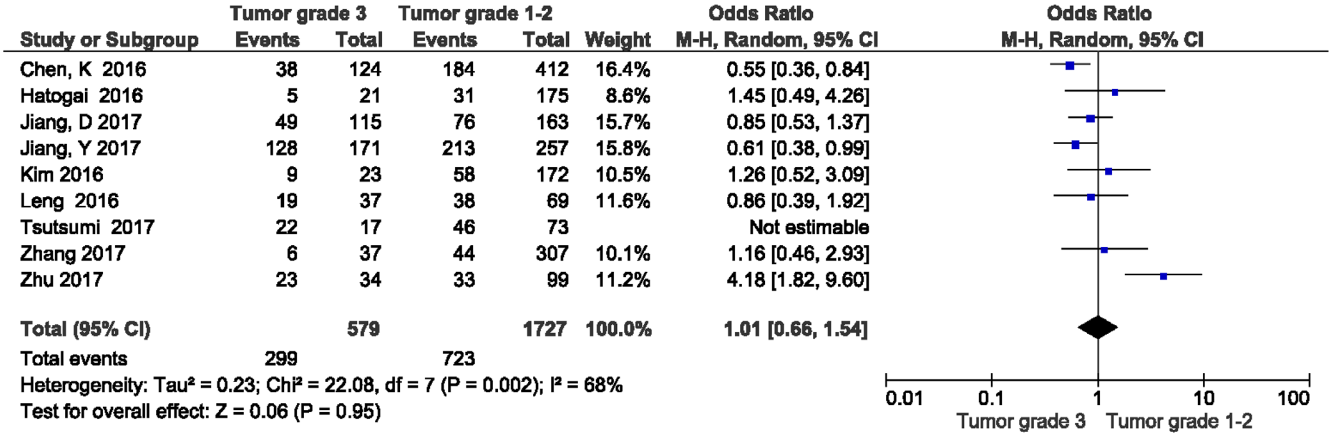

Supplementary Figure 2: Forest plot describing the association between PD-L1 expression and tumor differentiation.

Study or Subgroup	T3+T4 Events	T3+T4 Total	T1+T2 Events	T1+T2 Total	Weight	Odds Ratio M-H, Random, 95% CI
Chen, K 2016	165	416	57	120	22.7%	0.73 [0.48, 1.09]
Chen, L 2014	57	64	25	35	11.3%	3.26 [1.11, 9.54]
Hatogai 2016	29	164	7	32	13.2%	0.77 [0.30, 1.94]
Jiang, Y 2017	193	240	71	93	19.5%	1.27 [0.72, 2.26]
Tanaka 2016	35	112	18	68	17.6%	1.26 [0.65, 2.47]
Zhang 2017	39	305	11	39	15.7%	0.37 [0.17, 0.81]
**Total (95% CI)**		**1301**		**387**	**100.0%**	**0.96 [0.60, 1.53]**
Total events	518		189			

Heterogeneity: Tau² = 0.21; Chi² = 14.03, df = 5 (P = 0.02); I² = 64%
Test for overall effect: Z = 0.17 (P = 0.86)

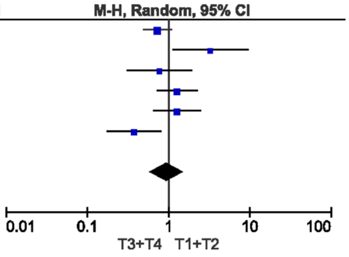

Supplementary Figure 3: Forest plot describing the association between PD-L1 expression and T stage.

Study or Subgroup	N+ Events	N+ Total	N- Events	N- Total	Weight	Odds Ratio M-H, Random, 95% CI
Chen, K 2016	107	314	115	222	13.8%	0.48 [0.34, 0.68]
Chen, L 2014	28	34	54	65	8.1%	0.95 [0.32, 2.84]
Chen, MF 2016	63	123	11	39	10.5%	2.67 [1.22, 5.84]
Hatogai 2016	26	145	10	51	10.2%	0.90 [0.40, 2.02]
Jiang, D 2017	58	133	67	145	13.0%	0.90 [0.56, 1.45]
Jiang, Y 2017	156	185	148	197	12.7%	1.78 [1.07, 2.97]
Tanaka 2016	45	127	8	53	10.0%	3.09 [1.34, 7.12]
Tsutsumi 2017	33	46	24	44	9.7%	2.12 [0.88, 5.07]
Zhang 2017	26	177	24	167	12.0%	1.03 [0.56, 1.87]
**Total (95% CI)**		**1284**		**983**	**100.0%**	**1.26 [0.80, 2.00]**
Total events	542		461			

Heterogeneity: Tau² = 0.36; Chi² = 37.73, df = 8 (P < 0.00001); I² = 79%
Test for overall effect: Z = 1.01 (P = 0.31)

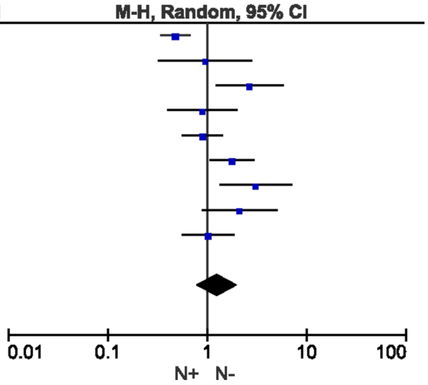

Supplementary Figure 4: Forest plot describing the association between PD-L1 expression and N stage.

Study or Subgroup	Metastasis + Events	Metastasis + Total	Metastasis - Events	Metastasis - Total	Weight	Odds Ratio M-H, Fixed, 95% CI
Chen, L 2014	6	6	76	93	2.3%	2.97 [0.16, 55.30]
Chen, MF 2016	35	68	39	94	48.8%	1.50 [0.80, 2.80]
Hatogai 2016	4	17	32	179	13.0%	1.41 [0.43, 4.62]
Zhang 2017	18	48	35	132	35.8%	1.66 [0.83, 3.35]
**Total (95% CI)**		**139**		**498**	**100.0%**	**1.58 [1.03, 2.42]**
Total events	63		182			

Heterogeneity: Chi² = 0.26, df = 3 (P = 0.97); I² = 0%
Test for overall effect: Z = 2.09 (P = 0.04)

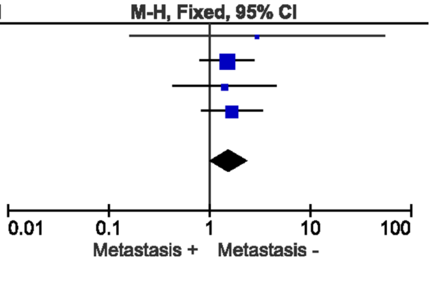

Supplementary Figure 5: Forest plot describing the association between PD-L1 expression and metastasis.

	III,IV		I,II			Odds Ratio
Study or Subgroup	Events	Total	Events	Total	Weight	M-H, Random, 95% CI
Chen, K 2016	101	280	125	256	18.6%	0.59 [0.42, 0.84]
Chen, L 2014	30	37	52	62	6.7%	0.82 [0.28, 2.39]
Hatogai 2016	25	138	11	58	9.9%	0.95 [0.43, 2.08]
Jiang, D 2017	53	110	72	168	15.5%	1.24 [0.76, 2.01]
Jiang, Y 2017	177	215	110	143	14.6%	1.40 [0.83, 2.36]
Kim 2016	30	75	37	125	13.1%	1.59 [0.87, 2.89]
Leng 2016	17	28	40	78	8.7%	1.47 [0.61, 3.54]
Zhang 2017	21	182	29	162	12.9%	0.60 [0.33, 1.10]
**Total (95% CI)**		**1065**		**1052**	**100.0%**	**0.99 [0.72, 1.38]**
Total events	454		476			

Heterogeneity: Tau² = 0.12; Chi² = 16.41, df = 7 (P = 0.02); I² = 57%
Test for overall effect: Z = 0.04 (P = 0.97)

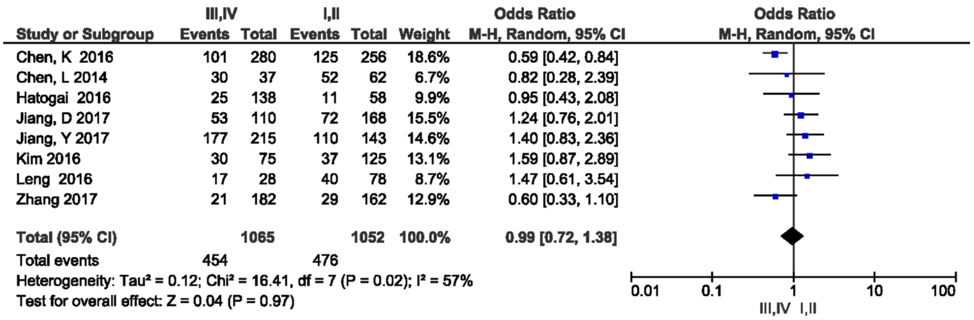

Supplementary Figure 6: Forest plot describing the association between PD-L1 expression and TNM stage.

	Present		Absent			Odds Ratio
Study or Subgroup	Events	Total	Events	Total	Weight	M-H, Fixed, 95% CI
Hatogai 2016	21	103	15	93	22.6%	1.33 [0.64, 2.77]
Jiang, D 2017	58	133	67	145	65.1%	0.90 [0.56, 1.45]
Tsutsumi 2017	35	49	22	41	12.3%	2.16 [0.90, 5.17]
**Total (95% CI)**		**285**		**279**	**100.0%**	**1.15 [0.81, 1.65]**
Total events	114		104			

Heterogeneity: Chi² = 3.19, df = 2 (P = 0.20); I² = 37%
Test for overall effect: Z = 0.78 (P = 0.44)

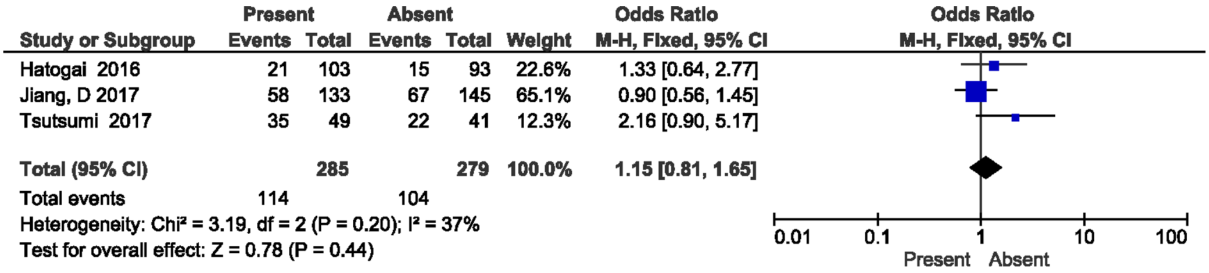

Supplementary Figure 7: Forest plot describing the association between PD-L1 expression and lymphatic invasion.

	Present		Absent			Odds Ratio
Study or Subgroup	Events	Total	Events	Total	Weight	M-H, Fixed, 95% CI
Hatogai 2016	29	172	7	24	28.1%	0.49 [0.19, 1.29]
Jiang, D 2017	25	51	100	227	51.5%	1.22 [0.66, 2.24]
Tsutsumi 2017	20	29	37	61	20.4%	1.44 [0.56, 3.69]
**Total (95% CI)**		**252**		**312**	**100.0%**	**1.06 [0.67, 1.67]**
Total events	74		144			

Heterogeneity: Chi² = 3.04, df = 2 (P = 0.22); I² = 34%
Test for overall effect: Z = 0.26 (P = 0.80)

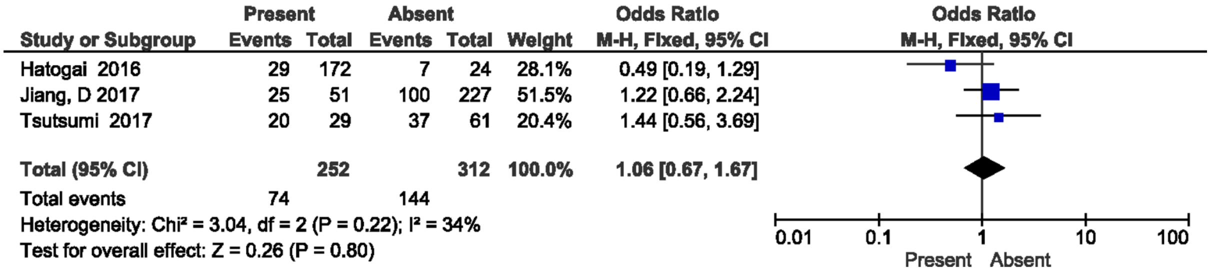

Supplementary Figure 8: Forest plot describing the association between PD-L1 expression and venous invasion.

	Smoke +		Smoke -			Odds Ratio
Study or Subgroup	Events	Total	Events	Total	Weight	M-H, Fixed, 95% CI
Chen, K 2016	166	403	56	133	49.6%	0.96 [0.65, 1.43]
Hatogai 2016	26	154	10	42	13.1%	0.65 [0.28, 1.48]
Jiang, D 2017	48	115	77	162	37.3%	0.79 [0.49, 1.28]
**Total (95% CI)**		**672**		**337**	**100.0%**	**0.86 [0.64, 1.14]**
Total events	240		143			

Heterogeneity: Chi² = 0.87, df = 2 (P = 0.65); I² = 0%
Test for overall effect: Z = 1.05 (P = 0.30)

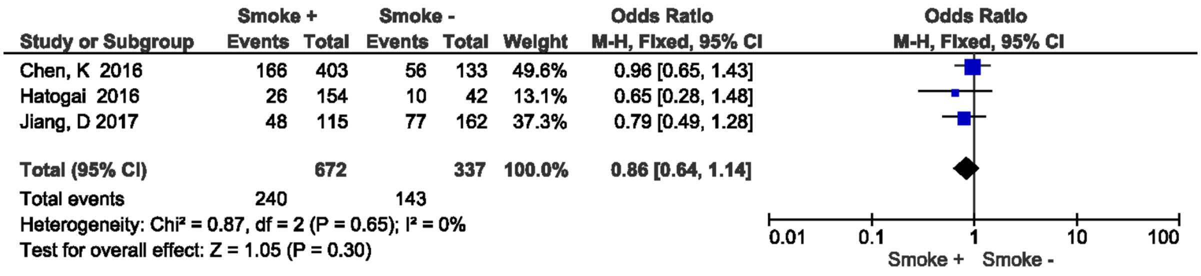

Supplementary Figure 9: Forest plot describing the association between PD-L1 expression and smoking.

Study or Subgroup	Alcohol + Events	Alcohol + Total	Alcohol - Events	Alcohol - Total	Weight	Odds Ratio M-H, Fixed, 95% CI
Chen, K 2016	157	382	65	154	84.4%	0.96 [0.65, 1.40]
Hatogai 2016	29	170	7	26	15.6%	0.56 [0.21, 1.45]
**Total (95% CI)**		**552**		**180**	**100.0%**	**0.89 [0.63, 1.27]**
Total events	186		72			

Heterogeneity: Chi² = 1.05, df = 1 (P = 0.30); I² = 5%
Test for overall effect: Z = 0.62 (P = 0.53)

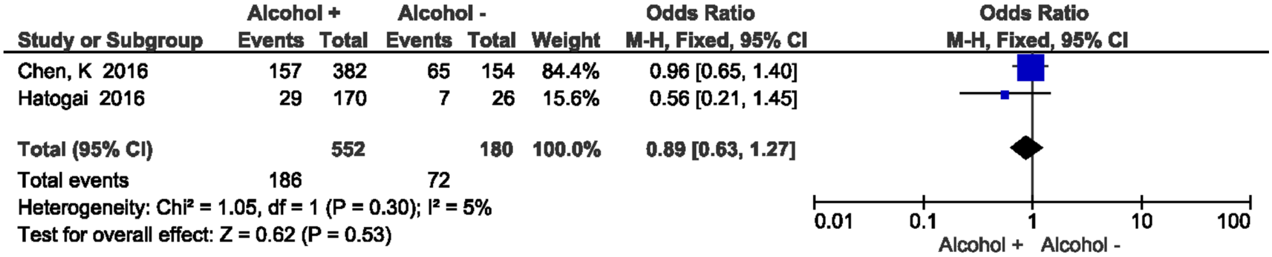

Supplementary Figure 10: Forest plot describing the association between PD-L1 expression and drinking.

Study or Subgroup	neoadjuvent + Events	neoadjuvent + Total	neoadjuvent - Events	neoadjuvent - Total	Weight	Odds Ratio M-H, Random, 95% CI
Chen, MF 2016	18	47	56	115	35.7%	0.65 [0.33, 1.31]
Kim 2016	8	20	59	180	29.2%	1.37 [0.53, 3.53]
Tanaka 2016	40	111	13	69	35.1%	2.43 [1.18, 4.97]
**Total (95% CI)**		**178**		**364**	**100.0%**	**1.28 [0.57, 2.92]**
Total events	66		128			

Heterogeneity: Tau² = 0.37; Chi² = 6.69, df = 2 (P = 0.04); I² = 70%
Test for overall effect: Z = 0.60 (P = 0.55)

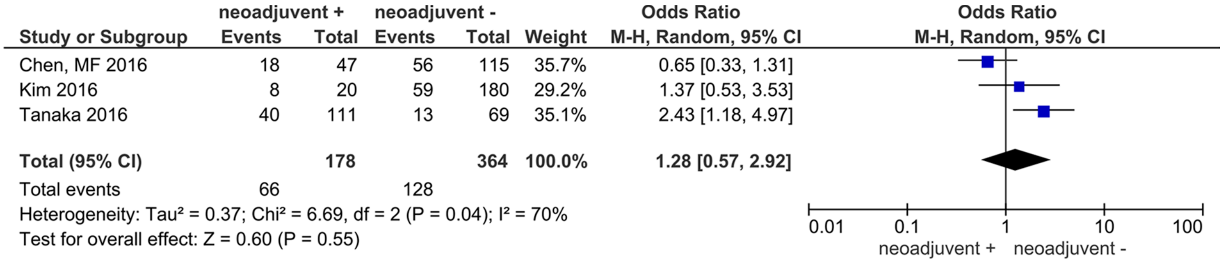

Supplementary Figure 11: Forest plot describing the association between PD-L1 expression and neoadjuvant treatment.
